# DNA Glycosylases in Noncancer Diseases: Oxidative Lesion Recognition, Repair Intermediates, and Tissue Responses

**DOI:** 10.3390/ijms27219915

**Published:** 2026-11-05

**Authors:** Su-Jin Kang, Yubin Ko, Subin Cho, Sunbok Jang

**Affiliations:** 1 College of Pharmacy, Dongduk Women’s University, Seoul 02748, Republic of Korea; sjkang@dongduk.ac.kr; 2 College of Pharmacy, Graduate School of Pharmaceutical Sciences, Ewha Womans University, Seoul 03760, Republic of Korea; koyubin@ewha.ac.kr; 3 Graduate Program in Innovative Biomaterial Convergence, Ewha Womans University, Seoul 03760, Republic of Korea; 4 College of Pharmacy, Graduate School of Industrial Pharmaceutical Science, Ewha Womans University, Seoul 03760, Republic of Korea; bin.some01@ewha.ac.kr; 5 Graduate Program in Regulatory Science Convergence, Ewha Womans University, Seoul 03760, Republic of Korea

**Keywords:** DNA glycosylases, oxidative DNA damage, BER, repair intermediates, inflammatory signaling, telomeric senescence, mtDNA repair, cardiometabolic disease, neurodegeneration

## Abstract

Oxidative DNA damage in noncancer tissues can perturb transcription, compromise telomere integrity, and impair mitochondrial function. DNA glycosylases initiate base excision repair (BER), but their biological consequences also depend on lesion recognition, chromatin residence, catalytic processing, and the persistence or resolution of abasic sites and strand breaks. This review integrates structural and kinetic evidence with experimental models of inflammation, telomeric senescence, cardiometabolic disease, neurodegeneration, and neural injury. Promoter-associated 8-oxoguanine DNA glycosylase 1 (OGG1) can either promote inflammatory transcription or restrict antiviral interferon expression in a locus-dependent manner. At oxidatively damaged telomeres in human fibroblasts, OGG1 and mutY DNA glycosylase (MUTYH) drive acute senescence through repair intermediates, whereas during repeated injury, OGG1 supports telomere maintenance and MUTYH-dependent arrest limits chromosome instability. Mitochondria-directed OGG1 expression improves metabolic outcomes in OGG1-deficient mice exposed to a high-fat diet. By contrast, vascular and neural phenotypes associated with Nei-like DNA glycosylase 3 (NEIL3) remain difficult to attribute to a defined initiating lesion. These findings emphasize distinguishing enzyme occupancy, catalytic processing, and completed repair. Therapeutic studies should define the relevant cell, substrate, compartment, and exposure period and assess tissue function directly alongside unresolved lesions and chromosome stability.

## 1. Introduction

The premise that increasing oxidative DNA repair will necessarily improve tissue function is appealing but overly simplistic. Inhibition of 8-oxoguanine DNA glycosylase 1 (OGG1) reduces airway inflammatory transcription in experimental systems [1,2], whereas mitochondria-directed OGG1 expression protects mice against multiple consequences of a high-fat diet [3]. At oxidatively damaged telomeres, suppressing OGG1- and mutY DNA glycosylase (MUTYH)-dependent repair initiation initially protects human fibroblasts from senescence, yet sustained damage reveals protective functions of both repair and checkpoint arrest [4]. These apparently divergent outcomes reflect distinct molecular perturbations and cannot be positioned along a simple continuum of beneficial versus detrimental glycosylase activity. A more informative question is which molecular state is altered within each tissue and whether the initiating lesion is ultimately resolved.

This distinction is particularly consequential in noncancer disease. Airway epithelial cells, vascular smooth muscle cells, hepatocytes, and aging fibroblasts can undergo substantial functional deterioration without the clonal selection that characterizes many cancer models. Oxidative damage can disrupt transcription, mitochondrial physiology, telomere replication, and innate immune responses through mechanistically distinct intermediates. A knockout phenotype can establish a requirement for a protein but simultaneously eliminates substrate recognition, catalytic activity, partner interactions, and functions across distinct cellular compartments. Likewise, an increased 8-oxo-7,8-dihydroguanine (8-oxoG) signal does not identify the underlying cellular process because its abundance reflects the combined effects of lesion formation and removal, DNA turnover, and the ability of the assay to capture particular DNA structures [5,6].

This review examines how lesion accessibility, base excision, and product release give rise to distinct outcomes across noncancer tissues [6]. Studies of promoters and telomeres demonstrate that enzyme occupancy or repair initiation can alter cellular state before repair is completed [1,4]. Accordingly, disease models are evaluated with respect to both the causal lesion and the responsible cell population, while short-term functional benefit is considered alongside genome stability under sustained stress. Although previous reviews have addressed base excision repair (BER) biochemistry, oxidative DNA damage, and OGG1-associated signaling, a key gap remains in integrating these processes within a common framework for interpreting noncancer disease phenotypes. Unlike reviews focused primarily on individual repair pathways, lesion types, or regulatory functions, this review distinguishes lesion recognition, regulatory occupancy, catalytic processing, repair intermediates, and completed repair as experimentally separable molecular states. Applying this framework across inflammatory, telomeric, cardiometabolic, vascular, and neurological disease models allows direct evidence of molecular mechanisms to be distinguished from disease associations for which the initiating lesion or causal repair step remains unresolved.

Meaningful comparison does not require equivalent coverage of every glycosylase. OGG1, MUTYH, Nei-like DNA glycosylase 1 (NEIL1), Nei-like DNA glycosylase 2 (NEIL2), Nei-like DNA glycosylase 3 (NEIL3), and NTH-like DNA glycosylase 1 (NTHL1) differ both in the chemistry they recognize and in the strength of evidence linking their molecular activities to noncancer outcomes. NTHL1 provides an informative model for evaluating repair completion in cells subjected to oxidative stress [7,8,9], whereas the most clearly resolved noncancer disease mechanisms currently involve OGG1 and MUTYH [1,2,3,4]. Evidence from biochemical systems or cancer-associated variants is therefore considered when it interrogates a transferable molecular step, rather than as direct evidence of a noncancer phenotype. Throughout this review, binding, base excision, repair completion, and tissue response are treated as distinct experimental observations whose causal relationships must be established directly [10,11,12].

This narrative review was informed by a targeted literature search using PubMed, Web of Science, and Google Scholar, supplemented by searches of relevant journal and publisher websites. The search covered publications from 2006 to September 2026 and was last conducted on September 25, 2026. Search terms included “DNA glycosylases”, “base excision repair”, “oxidative DNA damage”, “OGG1”, “MUTYH”, “NEIL1”, “NEIL2”, “NEIL3”, and “NTHL1”, combined with terms related to inflammation, telomeres, mitochondrial DNA, metabolic disease, atherosclerosis, and neurological disorders. Original studies were selected for their relevance to substrate recognition, DNA binding, catalytic activity, repair-intermediate processing, repair completion, or noncancer tissue outcomes. Biochemical, structural, and cancer-derived studies were also considered when they provided relevant mechanistic evidence, but were not treated as direct evidence of noncancer disease mechanisms. The literature was synthesized according to mechanistic relevance rather than through a formal systematic review or meta-analysis.

## 2. Lesion Recognition and Repair Coordination

### 2.1. Lesion Chemistry and Glycosylase Reaction State

OGG1 recognizes 8-oxoG paired with cytosine and excises the oxidized base, whereas MUTYH acts on a distinct substrate by removing adenine opposite 8-oxoG, thereby leaving the oxidized guanine for subsequent processing once the pairing context has been corrected. This division of labor suppresses oxidative mutagenesis but also means that a common endpoint, such as an altered 8-oxoG burden, can obscure fundamentally different initiating reactions. This distinction is particularly important at regulatory DNA and telomeres, where repair-generated abasic sites or nicks may exert more immediate functional effects than the original oxidized base. Accordingly, both the base pair presented to the glycosylase and the downstream product whose persistence may alter cellular state must be defined [4,11].

Human MUTYH variants provide a direct test of whether stable DNA binding necessarily reflects productive repair. Recent structural and functional analyses showed that the R241Q and N238S variants retain the iron–sulfur cluster and bind the mismatch tightly but lack detectable glycosylase activity under the reported assay conditions. A structure incorporating a transition-state mimic, together with associated simulations, implicated a hydrogen-bond network linking the cofactor region to catalytic Asp236. These findings demonstrate that DNA association, protein integrity, and productive catalysis can be experimentally dissociated, without implying that the same separation applies to every variant. A complementary genome-editing study quantified repair of an introduced 8-oxoG paired with adenine in isogenic human cells, providing a framework for comparing biochemical assignments with repair activity in a cellular context. Neither study alone establishes a noncancer signaling function for MUTYH; together, however, they illustrate how this possibility can be tested without equating protein knockout with catalytic loss [11,13].

OGG1 poses a related interpretive challenge at the earliest stage of lesion recognition. A recent study combining stopped-flow measurements with live-cell imaging linked conserved DNA-proximal residues to coordinated sampling of candidate base pairs [6]. Cryogenic electron microscopy (cryo-EM) and biochemical analyses of lesion-containing nucleosomes further showed that OGG1 can engage damaged DNA while shifting and partially detaching it from the histone core [14,15]. Thus, structural accessibility is conditional rather than guaranteed by the presence of an 8-oxoG:C pair. By comparison, in reconstituted nucleosomes, 8-oxoG opposite adenine retained a recognizable base-pair geometry at multiple positions, whereas MUTYH-dependent adenine excision was strongly suppressed within the nucleosome core and remained detectable near an entry or exit region [16]. In this system, restricted access rather than loss of the modified base limited repair initiation. A disease-associated locus could therefore retain an oxidized base while generating few abasic sites until chromatin architecture changes. Testing this possibility requires coordinated measurements of lesion abundance, MUTYH occupancy, and repair products at the same locus. These purified systems, however, do not establish that chromatin remodeling or transcription-factor activity produces a specific patient phenotype.

### 2.2. Alternative DNA Architectures and NEIL Selectivity

The NEIL proteins illustrate why classification within a common glycosylase family does not imply interchangeable substrate preferences. Comparative assays using oxidized telomeric quadruplexes showed that mouse NEIL3 and human NEIL1 processed selected hydantoin lesions, whereas the tested enzymes did not excise 8-oxoG from these structures. Mouse NEIL3 also removed thymine glycol in a sequence-dependent manner. Additional studies of promoter and telomeric quadruplexes demonstrated that DNA topology and ionic conditions influence hydantoin processing. Partner proteins further shape substrate presentation. A NEIL1 variant engineered to weaken its interaction with replication protein A (RPA) retained thymine-glycol excision on a purified duplex DNA substrate but failed to rescue a mild nucleotide excision repair reporter defect in NEIL1-depleted cells [17]. These findings represent well-defined biochemical observations rather than evidence that the lesions identified in vitro constitute the initiating substrates in atherosclerotic smooth muscle cells or senescing human telomeres. They nevertheless establish that lesion identity must be interpreted in conjunction with strand presentation and higher-order DNA structure [18,19].

Recruitment of NEIL1 following OGG1 depletion or TH5487 treatment indicates that access to damaged DNA can be redistributed, but recruitment alone does not establish equivalent base-excision activity [20]. NEIL2 raises the more specific question of which stage of the repair pathway is being captured. A structure of a mammalian NEIL2 ortholog bound to an abasic-site analogue revealed interdomain closure and a distinctive insertion contacting the complementary DNA strand. Biochemical assays further showed that NEIL2 preferentially processed abasic substrates relative to most oxidized bases tested and acted efficiently in single-stranded DNA contexts. Removal of the insertion impaired abasic-site cleavage in duplex DNA while exerting a substantially smaller effect on the corresponding single-stranded substrate [12]. These findings make DNA architecture and repair-intermediate processing central to the interpretation of NEIL2 activity, but they do not establish that the same reaction occurs at an inflammatory promoter. Recruitment of NEIL2 near the respiratory syncytial virus (RSV)-responsive interferon beta (IFN-β) promoter provides a defined experimental setting in which catalytic rescue, combined with locus-specific measurements of oxidized bases and abasic sites, could distinguish base excision from intermediate processing and regulatory occupancy [21].

Structural observations constrain the range of plausible reactions, but binding and cleavage assays must be integrated with cellular measurements before disease mechanisms can be assigned. The absence of activity on a purified quadruplex does not exclude repair following structural unfolding in cells. Table 1 therefore distinguishes experimentally tested substrates from proposed disease-relevant activities [6,12,14].

### 2.3. Repair Intermediates and Pathway Completion

Base excision initiates a repair pathway rather than completing it. An OGG1-generated abasic site requires further processing, and the resulting DNA discontinuity must subsequently be filled and ligated. Bifunctional glycosylases can additionally introduce strand-incision chemistry, generating termini that require processing by downstream enzymes. Recent structural and computational analyses of OGG1 and NTHL1 (also known as NTH1) resolved a conserved catalytic dyad and directly visualized a product-bound state of OGG1 [10]. These intermediate structures sharpen the mechanistic question of whether a therapeutic agent enhances base removal, facilitates product release, or affects both steps. At sensitive genomic loci, the immediate repair products are not merely transient biochemical intermediates, because a nick can disrupt replication or sustain checkpoint signaling [4].

NTHL1 provides a particularly clear example of the consequences of incomplete repair of oxidized pyrimidines. Recent mechanistic work identified catalytic features shared with OGG1 while clarifying how NTHL1 processes the abasic intermediate generated after base excision [10]. A separate study engineered an NTHL1 variant that retained glycosylase activity but exhibited markedly reduced apurinic/apyrimidinic (AP)-lyase activity. In cells subjected to acute oxidative stress, this variant persisted longer at damaged chromatin, competed with apurinic/apyrimidinic endonuclease 1 (APE1) for abasic substrates, and was associated with more persistent abasic sites and X-ray repair cross-complementing protein 1 (XRCC1) foci. These cells also showed increased sensitivity to the oxidative challenge [7]. Together, these findings distinguish the capacity to remove a damaged base from the capacity to advance repair toward completion. They establish repair-step coordination as a biologically consequential property of NTHL1 without demonstrating a specific cardiometabolic disease mechanism.

The molecular handoff following glycosylase activity is not an automatic consequence of base excision. APE1 recognizes and incises abasic sites and also processes obstructive 3′ termini generated by bifunctional glycosylases [32,33]. Single-molecule experiments using purified APE1 and DNA polymerase β (Pol β) resolved the sequence of protein association and dissociation at a preformed 5′-nick repair intermediate under conditions in which catalysis was suppressed. After Pol β joined an APE1–DNA complex, Pol β dissociated first in most events, whereas APE1 dissociated first in a minority of events classified as DNA handoff [34]. More recent imaging studies showed that APE1 locates abasic sites through a dynamic search process and subsequently forms a more stable association with the site [35]. This behavior represents a plausible kinetic checkpoint, because delayed APE1 access prolongs persistence of the abasic state, whereas incision before the next enzyme becomes available generates a nick. Neither residence time alone nor a reduction in intact 8-oxoG directly reports the rate at which repair is completed and the DNA strand is resealed.

Pol β removes the 5′-deoxyribose phosphate and fills the resulting single-nucleotide gap, whereas XRCC1 coordinates access of polymerase and ligase to strand-break intermediates. Disruption of the Pol β–XRCC1 interface altered recruitment to DNA damage and basal polymerase abundance in mice without compromising viability, demonstrating that a molecular handoff and a gross organismal endpoint need not change in parallel [36]. XRCC1 also coordinates poly(ADP-ribose) polymerase (PARP)-dependent recruitment at sites of DNA damage [37]. In purified systems, interactions of DNA ligase I and ligase IIIα with APE1 and Pol β influenced nick sealing, whereas unfilled gaps permitted aberrant ligation and small deletions [38,39,40]. These experiments directly support an intermediate-centered model of BER imbalance. However, findings obtained with defined substrates do not establish that the same deletion mechanism occurs at inflamed promoters or human telomeres.

Chromatin architecture further modifies the balance among these repair steps. APE1 cleaves an exposed nucleosomal abasic site far more efficiently than an occluded site [33]. Reconstituted nucleosomes likewise retain NTHL1-generated products that APE1 processes efficiently in naked DNA [32]. A broader nucleosome-based screen identified positions at which repair stalls during polymerase activity or ligation after initiation [41]. Because this screen used uracil as the initiating lesion, it should not be interpreted as a direct measurement of oxidative-base repair in diseased tissue. Cells lacking APE1 can engage slower alternative pathways, including NTHL1-dependent AP-site processing, but reporter repair in these cells does not demonstrate that such alternatives provide sufficient capacity during repeated oxidative injury [9]. Collectively, these studies establish local AP sites, nick termini, gap filling, and completed ligation as distinct experimental readouts when evaluating a proposed signaling role for a glycosylase.

The kinetics of molecular handoff have also been examined in systems directly relevant to OGG1 and MUTYH. Single-molecule imaging demonstrated transient association of the ultraviolet-damaged DNA-binding complex (UV-DDB) with OGG1 or APE1, facilitated dissociation from damaged DNA, and improved downstream BER reactions [22]. A subsequent study showed that UV-DDB increased MUTYH turnover and shortened its residence on an abasic-site product [26]. Experiments with single-strand-selective monofunctional uracil DNA glycosylase 1 (SMUG1) extended this principle to a different substrate, demonstrating a shorter DNA-bound lifetime in the presence of UV-DDB [42]. These observations establish partner-sensitive regulation of product handling under the tested conditions. They do not demonstrate that UV-DDB performs an equivalent regulatory function at every inflamed promoter or within mitochondria, but they identify residence time and molecular handoff as measurable alternatives to invoking a nonspecific repair imbalance.

Chromatin immunoprecipitation (ChIP) enrichment integrates several variables, including lesion abundance, chromatin accessibility, protein concentration, and binding lifetime. Single-molecule measurements [23], combined with mapping of lesions, abasic sites, strand breaks, and sealed repair products [43], can help distinguish persistent binding from repeated recruitment. When an inhibitor reduces occupancy, time-resolved measurements of protein binding, repair products, and protein abundance can further distinguish impaired entry from accelerated release or protein loss [1,22,23].

**Figure 1:**
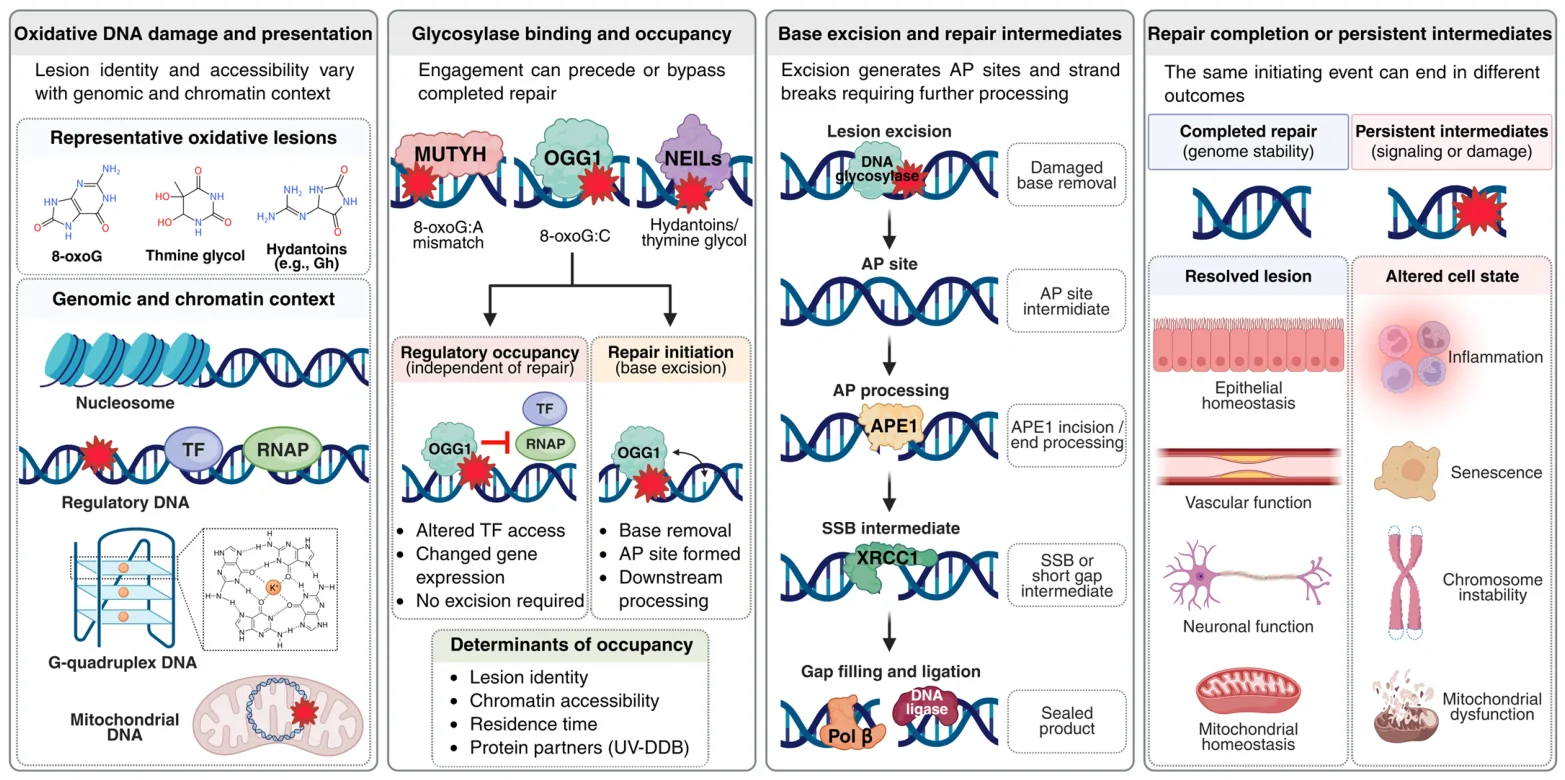
Molecular states from lesion recognition to tissue outcomes. Oxidative DNA lesions arise in distinct genomic and chromatin contexts, including nucleosomes, regulatory DNA, G-quadruplex structures, and mitochondrial DNA. Glycosylase engagement depends on lesion identity, DNA accessibility, residence time, and protein partners. Depending on the enzyme, substrate, and context, glycosylase engagement may lead to regulatory occupancy or repair initiation. Regulatory occupancy can influence transcription without completed repair, whereas base excision generates abasic (AP) sites and strand-break intermediates requiring downstream processing by APE1, XRCC1, DNA polymerase β (Pol β), and DNA ligase. Completed repair supports genome integrity and tissue homeostasis, whereas unresolved intermediates or prolonged regulatory occupancy may contribute to pathological cellular responses. Abbreviations: 8-oxoG, 8-oxo-7,8-dihydroguanine; AP, apurinic/apyrimidinic; APE1, apurinic/apyrimidinic endonuclease 1; Gh, guanidinohydantoin; LIG, DNA ligase; MUTYH, mutY DNA glycosylase; NEILs, Nei-like DNA glycosylases; OGG1, 8-oxoguanine DNA glycosylase 1; Pol β, DNA polymerase β; RNAP, RNA polymerase; SSB, single-strand break; TF, transcription factor; UV-DDB, ultraviolet-damaged DNA-binding protein complex; XRCC1, X-ray repair cross-complementing protein 1. Created in Biorender. Jang, S. (2026) https://BioRender.com/upk7mw4 (accessed on 11 October 2026).

Figure 1 integrates lesion identity and genome context with glycosylase engagement, regulatory occupancy, repair initiation, intermediate processing, and the divergent outcomes of completed versus unresolved repair.

## 3. Inflammatory and Antiviral Regulation

### 3.1. OGG1 Promoter Occupancy and Inflammatory Transcription

OGG1 provides the strongest experimental evidence for a glycosylase-associated regulatory state. The initial TH5487 study demonstrated that disrupting OGG1 engagement at guanine-rich inflammatory promoters reduced induced gene expression and inflammatory responses in mice [1]. A subsequent *Ogg1* S326C knock-in study provided a clearer separation between lesion removal and tissue response. Following inflammatory stimulation, the variant underwent reactive oxygen species (ROS)-dependent dimerization, exhibited impaired excision, remained associated with promoter-localized substrate, and coincided with increased nuclear factor κB (NF-κB) occupancy and more sustained airway inflammation. A small molecule that prevented DNA engagement by the variant attenuated the inflammatory phenotype [2]. Collectively, these observations support a promoter-associated regulatory mechanism without requiring OGG1 to function as a sequence-specific transcription factor.

The S326C findings are particularly informative because reduced catalytic activity coincided with greater inflammatory output. Protein removal, inhibition of DNA engagement, and persistence of a catalytically impaired protein on DNA represent distinct molecular states and therefore cannot be assumed to produce equivalent effects. This interpretation does not, however, imply that a single unmeasured kinetic parameter accounts for the entire knock-in phenotype. Substitution of Ser326 may also alter a phosphorylation-dependent response, while ROS-dependent oligomerization can influence processes beyond a single cleavage step. The study therefore establishes an enzyme-dependent relationship among molecular behavior, promoter occupancy, and inflammation. Assigning a unique causal role to slowed catalysis would require an additional perturbation that preserves the relevant DNA-binding properties and post-translational regulation while selectively altering catalytic chemistry [2]. Increased OGG1 occupancy at activated promoters could also partly reflect greater chromatin accessibility following transcriptional activation, rather than necessarily indicating that OGG1 binding precedes transcriptional activation. Time-resolved measurements of chromatin accessibility, OGG1 occupancy, and transcription at the same loci would help distinguish these possibilities.

Promoter-associated OGG1 is relevant beyond individual cytokine responses. In experimental lung injury, OGG1 and 8-oxoG at regulatory DNA contributed to fibrogenic transcription and airway remodeling, with TH5487 attenuating the reported response [24]. More recent work in kidney disease identified a nuclear association between OGG1 and phosphorylated Smad3 and showed that genetic or pharmacological interference with OGG1 attenuated renal fibrosis in mouse models [25]. These studies broaden the disease context of local OGG1-associated regulation but do not establish that promoter oxidation constitutes a stable epigenetic mark or that the same physical complex operates in both organs. A shared mechanistic prediction can instead be tested through matched measurements of lesion location, factor occupancy, transcriptional output, and histological injury within each model.

Fibrosis studies further illustrate why a tissue-level endpoint alone cannot identify the specific OGG1-containing complex that has been altered. In a bleomycin model, TH5487 also affected macrophage polarization and mitophagy-associated measures, introducing an immune-cell mechanism alongside epithelial or fibroblast transcriptional effects [44]. The relative contributions of these cell populations may also shift over the course of injury. A temporally controlled intervention restricted to a defined cell population could help distinguish direct alteration of a regulatory complex from secondary changes in tissue composition. The kidney and lung studies therefore provide a useful cross-organ comparison, but similar drug effects in two organs need not arise from identical lesion locations or protein partners. Within the present framework, these findings define testable mechanistic alternatives rather than supporting a single unified OGG1 pathway for fibrosis.

### 3.2. Interferon Regulation at Oxidatively Damaged Promoters

A uniformly proinflammatory model of OGG1 is insufficient at antiviral loci. In RSV-infected epithelium, OGG1 occupied oxidized regulatory DNA near interferon lambda (IFN-λ2/3), promoted binding of suppressive p50 homodimers, and restricted access of interferon regulatory factor 3 (IRF3), interferon regulatory factor 7 (IRF7), and RelA. Loss or inhibition of OGG1 increased epithelial IFN-λ expression. In a corresponding bronchiolitis model, TH5487 enhanced antiviral protection while reducing neutrophilia and immunopathology [45]. Thus, the intervention simultaneously enhanced a protective interferon program and reduced inflammatory injury. OGG1-dependent occupancy therefore operates within the transcription-factor network present at a specific locus and under a particular challenge, rather than imposing a uniform direction of regulation across immune genes.

NEIL2 provides a useful comparison at a distinct interferon locus. Following RSV infection, NEIL2 was recruited near an oxidatively damaged IFN-β promoter and limited early NF-κB-dependent transcription in airway epithelial cells [21]. The reported selectivity for IFN-β under these experimental conditions argues against assigning equivalent functions to all virus-responsive interferons or across the entire NEIL family. More broadly, one glycosylase may constrain a transcriptional program while another promotes expression at a different locus. A coherent mechanistic account of inflammatory regulation must therefore specify the lesion-bearing locus, the relevant transcription factors, and the temporal point at which DNA binding or processing alters their accessibility. Bulk cytokine measurements alone cannot distinguish among these alternatives.

Evidence that oxidized guanine itself can influence transcription independently of OGG1 or MUTYH places an important boundary on the reader model. A genome-wide mapping and RNA-sequencing study in glycosylase-deficient human cells identified associations between promoter-localized 8-oxoG and gene expression that persisted independently of both enzymes [5]. These correlations do not establish a single, universal, direct pathway from a specific oxidized base to every altered transcript. They do, however, demonstrate that enzyme-dependent transcription and lesion-associated transcription represent separable mechanistic propositions. Characterizing 8-oxoG as a programmable and heritable epigenetic mark would require evidence of controlled deposition, reproducible decoding, and inheritance, none of which can be inferred from oxidative stress and promoter occupancy alone.

### 3.3. Viral RNA Engagement and Mitochondrial DNA Signaling

NEIL2 also influences infection through an activity that cannot be explained solely by its role at the oxidatively damaged IFN-β promoter. In cultured cells, recombinant NEIL2 associated with the 5′ untranslated region of severe acute respiratory syndrome coronavirus 2 (SARS-CoV-2) RNA and reduced viral protein expression and infectious viral yield. In infected hamsters, lower NEIL2 expression was accompanied by greater DNA damage, although NEIL2-mediated viral restriction was not tested in vivo [29]. The study also reported reduced inflammatory expression and evidence of host genomic damage during infection. These findings support RNA engagement as an experimentally distinct mechanism of antiviral protection without demonstrating that an NEIL2-catalyzed DNA repair reaction mediates the RNA-dependent effect. This distinction is important because a family-wide model of glycosylases as readers of oxidized bases at promoters would obscure the involvement of a fundamentally different nucleic-acid substrate.

**Figure 2:**
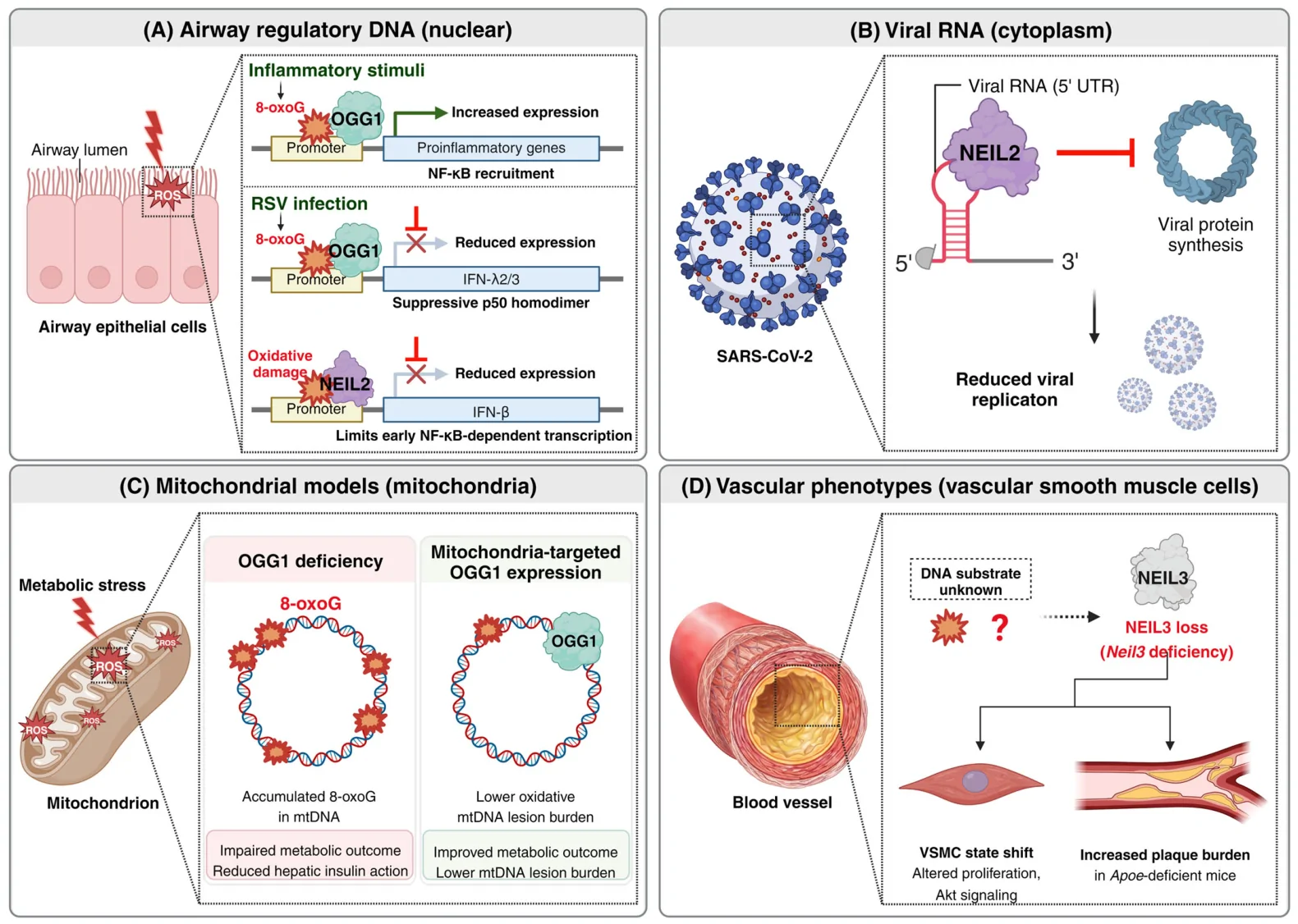
Glycosylase-associated responses across distinct substrates and cellular compartments. (**A**) In airway epithelial cells, OGG1 occupancy at oxidatively damaged inflammatory promoters is associated with increased NF-κB recruitment and proinflammatory gene expression, whereas OGG1 occupancy at RSV-responsive IFN-λ2/3 loci restricts interferon expression. NEIL2 recruitment near an oxidatively damaged IFN-β promoter similarly limits early NF-κB-dependent transcription. (**B**) NEIL2 association with the SARS-CoV-2 RNA 5′ untranslated region reduces viral protein expression and infectious viral yield in cultured cells, independently of a demonstrated DNA repair mechanism. (**C**) High-fat-fed *Ogg1*-deficient mice showed greater metabolic impairment than wild-type mice. In a separate transgenic comparison, mitochondria-targeted OGG1 expression in the knockout background reduced oxidative mtDNA damage and improved metabolic outcomes. (**D**) NEIL3 deficiency is associated with altered vascular smooth muscle cell behavior and increased plaque burden in *Apoe*-deficient mice, although the initiating DNA substrate remains unidentified. The panels represent distinct experimental settings rather than sequential steps of a common pathway. Abbreviations: 8-oxoG, 8-oxo-7,8-dihydroguanine; IFN, interferon; mtDNA, mitochondrial DNA; NEIL2, Nei-like DNA glycosylase 2; NEIL3, Nei-like DNA glycosylase 3; NF-κB, nuclear factor κB; OGG1, 8-oxoguanine DNA glycosylase 1; ROS, reactive oxygen species; RSV, respiratory syncytial virus; SARS-CoV-2, severe acute respiratory syndrome coronavirus 2; UTR, untranslated region; VSMC, vascular smooth muscle cell. Created in Biorender. Jang, S. (2026) https://BioRender.com/rl79lrq (accessed on 11 October 2026).

Mitochondrial integrity provides another pathway through which glycosylase activity may influence inflammatory phenotypes. Mitochondria-targeted OGG1 expression in aging mouse and cellular models was associated with reduced cytosolic mitochondrial DNA (mtDNA), lower stimulator of interferon genes (STING)-linked responses in the tested cells, and diminished hippocampal inflammatory signatures. Independent senescence and aging models have established that cytosolic mtDNA can activate cyclic GMP–AMP synthase (cGAS) and STING signaling [46,47]. Together, these findings define a testable pathway linking OGG1-dependent mitochondrial maintenance to inflammation, although those studies do not establish that loss of OGG1 initiated mtDNA release in their respective models. A separate pulmonary fibrosis study linked OGG1 inhibition to macrophage polarization and PTEN-induced kinase 1 (PINK1)-associated mitophagy. These experimental systems should therefore be connected directly rather than assumed to reflect a common mechanism. Oxidation of mtDNA does not itself identify the cytosolic receptor responsible for sensing it, and the STING-linked readouts reported in these models represent downstream responses rather than direct recognition of the modified base [44,48]. Figure 2 compares representative glycosylase-associated responses across airway regulatory DNA, viral RNA, mitochondrial metabolic models, and vascular smooth muscle cells, while highlighting the unresolved initiating substrate in the vascular NEIL3 setting.

## 4. Telomeric Damage and Senescence

### 4.1. Localized Telomeric Lesions and Repair-Associated Senescence

Telomeres provide a more experimentally controlled setting for examining repair-associated injury than exposure to a whole-cell oxidant. An engineered lesion-targeting system generated 8-oxoG preferentially at telomeres and induced rapid senescence in human cells without requiring substantial telomere shortening [49]. Lesion localization is critical because telomeric repeats, replication dynamics, and chromosome-end protection can produce responses to strand discontinuities that differ from those elicited by randomly distributed base lesions. These targeted experiments demonstrate that oxidative damage at chromosome ends can alter cell fate, but they do not imply that every marker of cellular senescence in aging tissues originates from telomeric damage. Rather, they provide a defined experimental setting in which the lesion, its processing, and the resulting checkpoint response can be examined separately.

In human fibroblasts subjected to telomere-targeted oxidative damage, OGG1 loss or inhibition and MUTYH deficiency each reduced acute senescence, with a greater effect observed after combined loss [4]. This response involved single-strand breaks, disrupted telomere replication, and DNA damage signaling, although abasic sites, nicks, and completed repair patches were not tracked separately over time [4]. Persistent oxidized bases may nevertheless become consequential during later or repeated exposure. These findings link repair initiation to acute senescence without establishing whether further enhancement of repair initiation would exacerbate the senescent response.

Distinguishing among an abasic site, a nick, and a completed repair patch is particularly important at telomeres because their biological consequences may emerge over different timescales. Short-lived repair intermediates can be compatible with successful BER in a nonreplicating cell, whereas the same intermediates may impede a replication fork or sustain a persistent damage response when they arise within a vulnerable telomeric structure. Available experiments support single-strand breaks as an important mediator of the acute response but do not quantify every downstream repair step in individual cells or demonstrate that a single glycosylase acts at every oxidized base within telomeres. A logical next experiment would combine targeted lesion generation with time-resolved measurements of abasic sites, nicks, replication progression, and restoration of telomere integrity within the same experimental system [4,49].

### 4.2. Senescence and Chromosome Stability During Sustained Telomeric Damage

The acute rescue described above changed meaning when oxidative injury was repeated. Under sustained telomere-targeted damage, OGG1-deficient fibroblasts showed impaired growth, fragile telomeres, and chromosome ends lacking a detectable telomeric signal. Earlier experiments in cancer-derived cells also connected persistent telomeric 8-oxoG and OGG1 deficiency to telomere loss and crisis [50]. More recently, XRCC1-deficient cells exhibited telomeric chromatin leakage, inflammatory signaling, and senescence, implicating a downstream strand-break scaffold in an additional chromosome-end response [51]. Although these models differ in cellular context, together they reveal an important limitation of interpreting the acute response in isolation. OGG1-dependent processing may contribute to early checkpoint activation while remaining necessary for telomere maintenance during recurrent injury. An intervention evaluated solely by its initial effect on senescence could therefore fail to capture this later protective requirement [4].

Combined loss of OGG1 and MUTYH further clarifies the distinction between continued proliferation and genomic stability. Double-deficient fibroblasts exhibited reduced senescence and continued to divide during repeated telomeric injury despite retaining unrepaired oxidized bases. After prolonged exposure, these cells also showed increased chromatin bridges and cytoplasmic DNA [4]. MUTYH-dependent arrest is therefore consistent with a protective checkpoint function in this model, even though it restricts cell proliferation. A reduction in senescent cells cannot consequently be interpreted as durable benefit without assessing the chromosomal integrity transmitted to subsequent cell generations. Whether the same balance operates in aging tissues remains unresolved, particularly in organs comprising both dividing and nondividing cell populations [4].

**Figure 3:**
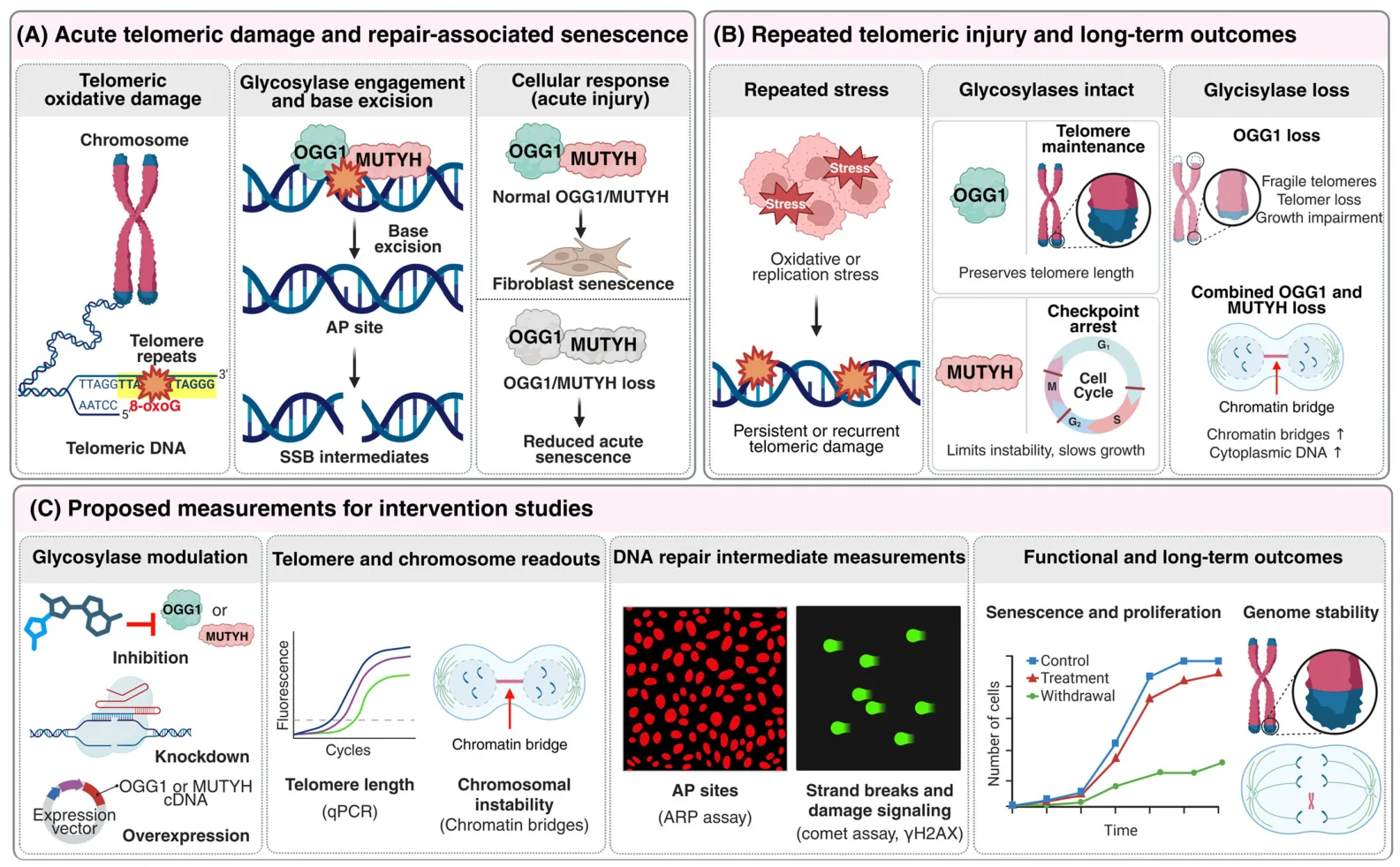
Acute and sustained consequences of telomeric oxidative damage and proposed measurements for intervention studies. (**A**) During acute telomeric oxidative injury, OGG1- and MUTYH-dependent repair initiation generates intermediates that contribute to fibroblast senescence. Loss of either glycosylase reduces this acute response. (**B**) Under repeated telomeric injury, OGG1 supports telomere maintenance, whereas MUTYH-dependent arrest limits chromosome instability. OGG1 loss is associated with telomere fragility and growth impairment, while combined OGG1 and MUTYH loss permits continued proliferation accompanied by chromatin bridges and cytoplasmic DNA accumulation. (**C**) Proposed intervention studies should assess telomere integrity, AP sites, strand breaks, DNA damage signaling, senescence, proliferation, and long-term genome stability across different treatment periods. Abbreviations: 8-oxoG, 8-oxo-7,8-dihydroguanine; AP, apurinic/apyrimidinic; ARP, aldehyde-reactive probe; cDNA, complementary DNA; MUTYH, mutY DNA glycosylase; OGG1, 8-oxoguanine DNA glycosylase 1; qPCR, quantitative polymerase chain reaction; SSB, single-strand break; γH2AX, phosphorylated histone H2AX. Created in Biorender. Jang, S. (2026) https://BioRender.com/jsl7tye (accessed on 11 October 2026).

Single and repeated telomeric injury should therefore be compared across defined periods of glycosylase modulation. Measurements of telomere loss, chromatin bridges, and cellular growth after treatment withdrawal could determine whether reduced senescence is accompanied by preservation of chromosome stability. XRCC1-dependent strand-break measurements could further link these outcomes to repair processes at chromosome ends [51]. Whether these telomeric findings extend to aging human organs remains unknown [4,49]. Figure 3 distinguishes the experimentally observed responses to acute and repeated damage from the additional readouts proposed for intervention studies.

## 5. Cardiometabolic and Neurological Diseases

### 5.1. NEIL3 in Vascular Disease and Substrate Uncertainty

Evidence linking NEIL3 to vascular disease begins with a physiological phenotype whose interpretation depends strongly on the experimental context. In high-fat-fed *Apoe*-deficient mice, additional loss of *Neil3* increased atherosclerotic plaque formation. These animals also exhibited altered plasma lipid profiles and hepatic triglyceride levels, while their macrophages showed reduced cholesterol efflux [31]. These findings introduce a systemic metabolic pathway alongside any effect occurring directly within the vessel wall. Because *Neil3* was deleted throughout the animal, the increased plaque burden cannot be attributed to a specific vascular cell population. The study therefore establishes a role for NEIL3 in the response to this dietary challenge while leaving the relative contributions of individual tissues unresolved [31].

A subsequent study provided a complementary experimental context. In chow-fed *Apoe*-deficient mice, *Neil3* loss increased plaque development without a detectable alteration in systemic lipid levels [30]. Analyses of vascular smooth muscle cells revealed increased proliferation, lipid accumulation, and expression of macrophage-associated features. Silencing *NEIL3* in primary human aortic smooth muscle cells further linked the altered proliferative phenotype to Akt signaling. Together, these observations support a vascular cell response that cannot be explained solely by the plasma lipid differences observed in the high-fat study [31]. However, they do not demonstrate direct binding between NEIL3 and Akt or establish that a defined oxidized DNA lesion initiates the observed cell-state transition.

The negative damage measurements are equally important for evaluating the underlying mechanism. The high-fat study did not detect a genotype-dependent increase in bulk oxidative DNA lesions or differences in DNA damage at the three hepatic promoter regions examined [31]. Similarly, the chow-fed study reported a vascular smooth muscle phenotype without a corresponding broad increase in measured DNA damage [30]. These findings constrain models based on widespread lesion accumulation. However, they do not exclude a transient repair intermediate or a lesion restricted to a small vascular cell population, because neither study resolved repair flux across every candidate locus. Increased NEIL3 expression in human plaques adds clinical relevance, although both plaque composition and local stress could influence this measurement. The mechanistic relationship between NEIL3 enzymatic activity and the observed Akt-associated phenotype therefore remains unresolved.

Cell-specific deletion and restoration of NEIL3 in smooth muscle cells, combined with parallel perturbations in liver and myeloid compartments, could help distinguish vascular from systemic effects. Rescue with a catalytically impaired variant would require verification of its expression, localization, and relevant molecular interactions. Candidate lesions and repair intermediates could then be evaluated alongside Akt activity and plaque composition.

### 5.2. Metabolic Phenotypes and Mitochondrial Repair

The metabolic evidence imposes distinct constraints on causal inference. In an early whole-body knockout study, *Neil1*-deficient mice developed obesity, abnormal circulating lipid profiles, hepatic steatosis, and a tendency toward hyperinsulinemia, accompanied by increased mtDNA damage and deletions [27]. A subsequent study reported variable phenotypic penetrance and accelerated high-fat-diet-associated adiposity, underscoring the influence of age, diet, and genetic background [28]. Mitochondrial lesions could contribute to disease initiation, accumulate secondary to metabolic dysfunction, or participate in a self-reinforcing cycle. These knockout studies do not distinguish among these temporal sequences or establish that an endogenous hepatic NEIL1 substrate constitutes the initiating lesion. Their principal strength is the identification of a reproducible physiological vulnerability whose causal direction requires resolution through targeted rescue experiments.

Studies of mitochondria-targeted OGG1 narrow one aspect of this causal problem. A 2024 investigation assessed hepatic insulin action using a hyperinsulinemic–euglycemic clamp in high-fat-fed wild-type and *Ogg1*-deficient male mice and found greater diet-associated metabolic impairment in the deficient animals. In a related but experimentally distinct transgenic comparison, expression of mitochondrial human OGG1 in the knockout background reduced oxidative mtDNA damage in the liver and improved several metabolic outcomes, accompanied by changes in gluconeogenic gene expression and oxidative phosphorylation proteins [3]. The clamp and transgenic findings should therefore be interpreted as separate experimental comparisons. The rescue experiment demonstrates that organelle-directed OGG1 expression can improve a metabolic phenotype, while leaving unresolved whether increased completion of mitochondrial BER alone accounts for this benefit.

NTHL1 provides a distinct cellular response within this mitochondrial framework. In human cells lacking *NTHL1*, mtDNA lesions accumulated despite increases in mtDNA copy number, oxidative phosphorylation proteins, and respiratory activity. These cells also exhibited greater resistance to the mitochondrial stressor examined. Mitochondria-directed NTHL1 reintroduction and reactive oxygen species scavenging reversed components of this response, supporting an organelle-dependent adaptive mechanism [8]. The direction of this short-term cellular phenotype differs from the metabolic improvement observed after mitochondrial OGG1 expression in diet-challenged mice. Because the models, exposures, and endpoints differ substantially, these findings do not support opposing therapeutic strategies for metabolic disease. Rather, they illustrate why mitochondrial lesion burden, respiratory performance, and tissue health must be evaluated independently before glycosylase modulation is characterized as uniformly beneficial or detrimental [3,8].

Mitochondrial BER presents a distinct problem of reaction completion. Mitochondrial APE1 can process abasic DNA, whereas polymerase γ and ligase III contribute to downstream DNA synthesis and sealing. Structural biochemical studies assign exonuclease G (EXOG) robust 5′-deoxyribose phosphate removal activity [52]. A subsequent reconstitution study showed that EXOG can process displaced single-stranded flaps and support polymerase γ and ligase III during mitochondrial DNA synthesis [53]. These defined substrates and purified reactions establish biochemical capabilities but do not identify the rate-limiting step in an obese liver. Differentiated neuronal and muscle cells also exhibit distinct flap-processing capacities in mitochondrial extracts [54]. Polymerase γ proofreading is itself essential for mitochondrial genome integrity, although the cited structural study examined replication rather than directly measuring BER flux [55]. Accordingly, metabolic studies should assess completed mtDNA repair and respiratory function alongside the lesions entering the repair pathway. Table 2 summarizes the experimental models, exposure conditions, key observations, and limits of causal inference in studies of NEIL1, OGG1, and NTHL1.

### 5.3. Neurodegeneration, Neural Injury, and Repair Heterogeneity

The brain imposes distinct constraints on repair-completion models because dividing neural precursors, long-lived neurons, and activated microglia may respond differently to the same repair intermediate. In a male mouse model exposed to the Parkinsonian toxin 1-methyl-4-phenyl-1,2,3,6-tetrahydropyridine (MPTP), *Neil1* deficiency increased nigrostriatal dopaminergic degeneration and DNA damage signals [56]. In another mouse model, MutT homolog 1 (MTH1) and OGG1 jointly limited oxidized-base accumulation and Alzheimer-like phenotypes [57]. These studies implicate oxidative DNA damage responses in neuronal vulnerability, but neither identifies a lesion sequence whose excision alone accounts for the complete behavioral phenotype. Oxidative DNA lesions can also contribute to neural pathology independently of glycosylase activity. A 2023 study linked oxidation-induced transcriptional mutagenesis of α-synuclein to aggregation-associated outcomes [58]. This mechanism should not be interpreted as evidence that OGG1 inhibition would correct α-synuclein production.

The potentially deleterious consequences of BER initiation are as important as those arising from insufficient repair. An experimental model of oxidative brain injury identified MUTYH-dependent processing of 8-oxoG as a contributor to neuronal degeneration [59]. In an Alzheimer-like mouse model, MUTYH also contributed to microglial activation and impaired neurogenesis [60]. Conversely, Pol β haploinsufficiency increased DNA damage and exacerbated neurodegenerative and cognitive phenotypes in a triple-transgenic Alzheimer disease (3xTgAD) cross [61]. These experiments involve distinct injury mechanisms and cell populations. Collectively, they indicate that limiting a repair-initiating enzyme may suppress a harmful intermediate in one model, whereas reducing downstream repair capacity may aggravate pathology in another. Neither comparison supports a broad clinical recommendation to inhibit or activate BER throughout the brain.

NEIL3 further illustrates the distinction between modifying a disease phenotype and identifying the causal base lesion. In an Alzheimer-like mouse model, NEIL3 deficiency produced age- and sex-dependent effects on amyloid pathology, adult neurogenesis, and memory [62]. Additional mouse studies linked NEIL3 to hippocampal progenitor responses and WNT-associated behavioral pattern separation [63], network dynamics involving parvalbumin-positive interneurons [64], and neurogenesis during the clinical phase of prion disease [65]. These studies establish multiple neural phenotypes but do not place a defined oxidized base upstream of each response. A 2025 investigation of OGG1 and MUTYH linked cognitive gene expression to polycomb repressive complex 2 (PRC2)-associated chromatin and DNA methylation changes in neurons and glia [66]. This finding supports a regulatory connection, although an individual 8-oxoG-containing promoter has not been established as a universal driver of these epigenetic effects.

Compartment-specific repair offers a tractable strategy for dissecting this heterogeneity. Mitochondrial OGG1 expression reduced cytosolic mtDNA and neuroinflammatory readouts during aging [48], whereas APE1 perturbation in a rat subarachnoid hemorrhage model altered measures of mitochondrial damage and neuronal injury [67]. The latter represents an acute cerebrovascular injury model rather than evidence for disease-modifying treatment of Alzheimer’s or Parkinson’s disease. Broad cGAS–STING blockade improved inflammatory and neurological measures in aging mice [47], but it did not establish an OGG1-dependent initiating lesion. Establishing a direct causal sequence requires cell-resolved measurements of lesions and repair intermediates, restoration of a defined repair step, and a matched neural functional endpoint. This framework would also determine whether a short-term reduction in inflammation leaves persistent oxidized bases or unresolved strand breaks in long-lived neurons.

Table 3 summarizes the principal noncancer disease models discussed above and distinguishes the experimentally observed findings from the causal steps that remain untested.

## 6. Therapeutic Modulation

### 6.1. Therapeutic Targeting of Defined Molecular States

Therapeutic design should begin by identifying the molecular state responsible for the measured phenotype. TH5487 interferes with OGG1 engagement of lesion-containing DNA and has reduced inflammation in preclinical respiratory and fibrotic models [1,24,25]. This mechanism differs fundamentally from suppressing bond cleavage while allowing the protein to remain associated with regulatory DNA. The S326C knock-in links this combination of impaired catalysis and persistent DNA association to sustained inflammation, but it does not separate catalytic slowing from the effects of the altered phosphorylation site and ROS-dependent dimerization; thus, the comparison establishes an enzyme-dependent association rather than a demonstrated catalytic mechanism [2]. This intervention also differs from reducing organellar 8-oxoG through mitochondria-targeted enzyme expression [3]. Accordingly, the therapeutic target should be defined as a specific molecular interaction within a particular tissue and linked to a measurable functional response. In the brain, this requirement additionally entails distinguishing neuronal survival from microglial activation and progenitor renewal [56,60,61]. Moreover, the same inhibitor can alter detrimental inflammatory transcription and protective antiviral interferon responses in opposite directions at different loci, necessitating joint assessment of these outcomes in infection-related indications [45].

OGG1 activation represents an experimentally distinct therapeutic strategy. Small molecules with proposed allosteric mechanisms increased selected OGG1 reactions in vitro and improved mitochondrial readouts in cellular models subjected to oxidative stress. Subsequent studies using nucleobase catalysts and organocatalytic approaches investigated acceleration of product-associated steps under defined biochemical conditions. More recent structural analyses captured OGG1 bound to a product and the agonist F51, supporting enhanced enzyme turnover through facilitated product release. Differences in assay conditions, ligands, and affected reaction steps should therefore be preserved rather than subsumed within a single activator category. Increased biochemical product formation does not establish that APE1, polymerase, and ligase complete BER more rapidly in the liver [34,38,40], nor does it demonstrate that an activated enzyme reaches the mitochondrial compartment required for metabolic protection [10,68,69].

An informative comparator design would independently perturb lesion recognition, catalysis, and product release and then quantify the original base together with resulting abasic sites and strand-break products over time. Inflammatory promoter occupancy, mitochondrial lesion burden, and telomere integrity require distinct assays of target engagement. A screen optimized exclusively for faster cleavage could identify compounds that increase unresolved repair intermediates, whereas a compound that prevents DNA engagement might suppress transcription while simultaneously delaying necessary repair. Separation-of-function alleles and cell-level substrate reporters provide valuable orthogonal controls, although neither can substitute for studies of tissue distribution, repeated exposure, and recovery following drug withdrawal. This experimental design is supported by the observed dissociation between protein binding and MUTYH catalysis and by direct evidence of partner-dependent glycosylase turnover [4,11,13,26].

### 6.2. Therapeutic Assessment and Genome Stability

Figure 3 illustrates why intervention studies should evaluate acute senescence together with genome stability during sustained telomeric injury. Acute suppression of OGG1- or MUTYH-dependent repair initiation reduced damage-associated senescence in fibroblasts, whereas sustained damage revealed impaired growth in the absence of OGG1 and a stabilizing role for MUTYH-dependent arrest. A transient inflammatory benefit alone cannot establish that prolonged systemic inhibition preserves chromosome stability. XRCC1-deficient cells provide an additional rationale for assessing telomeric chromatin leakage and downstream repair capacity [51]. Conversely, tissue-directed augmentation may be beneficial even if broad stimulation increases strand breaks at vulnerable loci. Dose, cellular compartment, and treatment interval should therefore be varied within the same model, and functional benefit should be evaluated alongside accumulated lesions, repair intermediates, and chromosome-end integrity before a long-term therapeutic indication is advanced [2,4,50].

Circulating oxidation products are insufficient as sole pharmacodynamic markers. A urinary nucleoside such as 8-oxo-7,8-dihydro-2′-deoxyguanosine (8-oxodG) is chemically distinct from a free oxidized base, and neither measurement localizes damage to a promoter, mitochondrion, or telomere. Changes in abundance may reflect lesion formation, release, repair, or disposal rather than completed repair flux. Direct nanopore detection of 8-oxodG offers a potential locus-sensitive measurement, but its performance must be established within the specific tissue and experimental context in which it is applied [70]. A more discriminating strategy would link verified target engagement to a mechanism-specific molecular readout and a disease-relevant functional outcome within the same model. Despite substantial preclinical progress, clinical efficacy of glycosylase-targeted therapies for the noncancer diseases discussed here has not yet been established.

Translation to human disease also requires careful interpretation of genetic and tissue-level evidence. An OGG1 variant with documented biochemical and promoter-associated behavior provides a mechanistically informative human allele, but a phenotype observed in a mouse knock-in model does not constitute evidence of therapeutic efficacy in human variant carriers [2]. Increased NEIL3 expression in atherosclerotic plaques may reflect differences in cellular composition, while reported associations between BER polymorphisms and fatty liver may be sensitive to sampling and confounding [71]. Primary human cells reduce some species-related limitations but cannot reproduce the duration and complexity of vascular or metabolic disease. A prospective translational program should therefore stratify tissues or genetic variants according to the reaction state actually demonstrated and predefine both efficacy and genome-maintenance outcomes. This approach is more informative than selecting an inhibitor or activator solely because the target protein is upregulated in diseased tissue.

## 7. Conclusions and Future Perspectives

We propose that the most informative unit of explanation in noncancer disease is a lesion–glycosylase–product state defined within a specific cell type and subcellular compartment. A glycosylase may remain bound to its substrate without prompt excision, whereas successful excision can generate repair intermediates whose biological consequences depend on subsequent pathway completion. The OGG1 S326C variant exemplifies this distinction by combining reduced excision with prolonged promoter occupancy and enhanced inflammatory output. At telomeres, OGG1- and MUTYH-dependent processing promotes acute senescence, whereas sustained damage reveals protective roles for repair and checkpoint arrest in maintaining chromosome stability. Thus, glycosylase abundance or bulk 8-oxoG levels provide incomplete proxies for tissue benefit. Enzyme residence time, repair-product formation, and completed repair should instead be considered distinct causal variables.

This framework provides a discriminating approach for evaluating vascular, metabolic, and neural disease models. A repair-based mechanism should identify the endogenous lesion and demonstrate that altered catalytic processing or downstream sealing changes the phenotype in the relevant cell type. An NTHL1 variant that accumulates abasic products after base removal, together with direct measurements of APE1–Pol β coordination, illustrates why repair-intermediate handling should be incorporated into this assessment. By contrast, the initiating NEIL3 substrate in atherosclerosis and the cell-specific reactions underlying several neural phenotypes remain unresolved. Separation-of-function rescue combined with locus-specific lesion mapping, intermediate quantification, and matched functional endpoints could distinguish altered repair flux from regulatory occupancy. A phenotype associated with altered binding but no measurable change in local repair products would favor investigation of a regulatory mechanism, whereas a phenotype that tracks completed repair would support a repair-dependent explanation.

Therapeutic studies should likewise be guided by the implicated mechanism and duration of exposure. OGG1 inhibition can reduce acute inflammatory responses, whereas mitochondria-directed OGG1 expression improves metabolic measures in a distinct experimental context. Neither observation establishes safety during sustained oxidative stress. A therapeutically informative intervention should define the target tissue, reaction state, and treatment interval and then evaluate functional improvement alongside retained lesions, abasic sites, strand breaks, and chromosome stability. Under this framework, reductions in cytokine production or senescent-cell abundance represent measured responses rather than evidence of durable genome protection. Selective glycosylase modulation becomes most compelling when it resolves a harmful molecular state within the intended compartment while preserving protective repair and checkpoint functions elsewhere.

## Figures and Tables

**Table 1:** Experimentally defined molecular and reaction states of DNA glycosylases across model systems and their relevance to noncancer disease. Measured biochemical, structural, and cellular activities are distinguished from evidence obtained in noncancer disease models.

Enzyme	Substrate and System	Measured Reaction State	Evidence (Molecular /Disease)	Noncancer Disease Status
OGG1	8-oxoG:C; purified protein, NCP, nuclear extract	Base-pair sampling during lesion search [6]; NCP engagement with partial DNA detachment [14]; UV-DDB-assisted dissociation [22]; differences in DNA-bound lifetime between purified protein and nuclear extract [23]	B, C /A, C	Promoter-associated state in airway and fibrotic models [1,2,24,25]; lesion-linked transcription persists in the absence of OGG1 or MUTYH [5]
MUTYH	A opposite 8-oxoG; duplex, NCP, isogenic human cells	Mismatch binding without excision by R241Q and N238S variants [11]; repair of a defined 8-oxoG:A lesion in cells [13]; excision blocked within the nucleosome core but permitted near DNA entry or exit sites [16]; UV-DDB-mediated shortening of product association [26]	B, C /C	Repair initiation contributes to acute telomeric senescence, whereas MUTYH-dependent arrest limits instability during sustained damage [4]
NEIL1	Hydantoins in telomeric and promoter quadruplexes; OGG1-depleted or TH5487-treated cells	Topology-dependent hydantoin removal [18,19]; recruitment to damaged DNA following OGG1 loss or inhibition [20]	B, C /A	Obesity and hepatic steatosis in whole-body knockout models, with dependence on age, diet, and genetic background [27,28]; endogenous initiating lesion remains unidentified
NEIL2	Abasic-site analogue; oxidatively damaged IFN-β promoter; SARS-CoV-2 5′ UTR RNA	Preference for product-like abasic DNA in the liganded structure [12]; promoter recruitment associated with restriction of early NF-κB-dependent IFN-β transcription [21]; RNA association associated with restricted viral replication [29]	B, C /A, C	Antiviral protection in infection models [29]; relative contributions of DNA catalysis and RNA binding remain unresolved
NEIL3	Structured DNA, mouse enzyme; *Apoe*-deficient mice; human aortic SMC	Sequence-sensitive removal of hydantoins and thymine glycol from quadruplex substrates [18,19]	B (ortholog) /A, C	Increased plaque burden associated with altered lipid handling and SMC state [30,31]; initiating substrate remains unassigned
NTHL1	Oxidized pyrimidines, thymine glycol	Oxidized-pyrimidine excision and AP-lyase activity [7,10]; product accumulation when downstream transfer is delayed [7,32]; mitochondrial stress adaptation [8]	B, C /C	Cellular oxidative and mitochondrial phenotypes [7,8]; tissue-level noncancer disease mechanism remains unassigned

Notes: Citations are provided with the specific observations they support. In vitro substrate specificity should not be interpreted as evidence of the same reaction in vivo. Evidence classes are presented separately for molecular findings and noncancer disease-related observations, before and after the slash, respectively: B, biochemical or structural studies; C, intact-cell studies; A, animal models. These classifications distinguish evidence for molecular reaction states from evidence for associated cellular or disease phenotypes. The NEIL1 cellular observation reflects recruitment to damaged DNA and does not by itself establish catalytic activity. The cited NEIL3 biochemical studies used mouse NEIL3 and therefore do not establish equivalent substrate chemistry for the human enzyme. The reported NTHL1 cellular phenotypes do not establish a tissue-level mechanism in noncancer disease. Abbreviations: 8-oxoG, 8-oxo-7,8-dihydroguanine; AP, apurinic/apyrimidinic; IFN-β, interferon beta; MUTYH, mutY DNA glycosylase; NCP, nucleosome core particle; NEIL1/2/3, Nei-like DNA glycosylases 1/2/3; NF-κB, nuclear factor κB; NTHL1, NTH-like DNA glycosylase 1; OGG1, 8-oxoguanine DNA glycosylase 1; SARS-CoV-2, severe acute respiratory syndrome coronavirus 2; SMC, smooth muscle cell; UTR, untranslated region; UV-DDB, ultraviolet-damaged DNA-binding protein complex.

**Table 2:** Comparison of experimental models and causal evidence for glycosylase-associated mitochondrial and metabolic phenotypes.

Glycosylase	Experimental Model and System	Exposure Conditions	Key Observations	Causal Interpretation and Limitations	Ref.
NEIL1	Whole-body *Neil1*-deficient mice; systemic and hepatic analyses	Age-dependent phenotype under standard chow feeding	Obesity, dyslipidemia, hepatic steatosis, and increased mtDNA damage and deletions	Establishes an association between NEIL1 deficiency and metabolic dysfunction, but does not identify the initiating tissue or endogenous lesion	[27]
	Whole-body *Neil1*-deficient mice; dietary challenge and hepatic analyses	HFD for 5 or 20 weeks; glucose tolerance assessed at 16 weeks in the long-term cohort	Increased adiposity and hepatic steatosis; impaired glucose tolerance and reduced hepatic mtDNA content after prolonged HFD exposure	Demonstrates diet-dependent metabolic vulnerability, but does not establish whether mitochondrial damage initiates or follows metabolic dysfunction	[28]
OGG1 deficiency	High-fat-fed wild-type and *Ogg1*-deficient male mice; hepatic insulin-action assessment	HFD for 16 weeks	Greater diet-associated metabolic impairment and reduced hepatic insulin action in *Ogg1*-deficient mice	Supports a contribution of OGG1 to metabolic regulation, but does not identify the responsible tissue-specific repair mechanism	[3]
Mitochondrial OGG1 expression	*Ogg1*-deficient mice expressing mitochondria-targeted OGG1; hepatic and systemic metabolic analyses	HFD for 16 weeks; separate transgenic comparison	Reduced oxidative mtDNA damage in the liver and improved metabolic outcomes	Supports a protective effect of mitochondria-targeted OGG1 expression, but does not establish that increased completion of mitochondrial BER alone mediates the benefit	[3]
NTHL1	NTHL1-deficient human cell models; mitochondrial functional and stress-response analyses	Genetic deficiency; MPP^+^ treatment for 72 h in stress-resistance assays	Increased mtDNA lesions, mtDNA copy number, oxidative phosphorylation proteins, and respiratory activity; enhanced resistance to mitochondrial stress	Supports a mitochondria-dependent adaptive response, but does not establish protection against metabolic disease in vivo or demonstrate increased completed repair flux	[8]

Notes: The NEIL1 studies include age-dependent phenotypes under standard feeding conditions and separate short- and long-term HFD challenges. In the long-term HFD experiment, glucose tolerance was assessed after 16 weeks, although the feeding period extended to 20 weeks. The OGG1 deficiency and mitochondria-targeted OGG1 expression findings represent separate experimental comparisons. The 72 h MPP^+^ exposure applies specifically to the NTHL1 stress-resistance assays. Differences in experimental models, exposure conditions, and measured outcomes preclude direct comparison of therapeutic effects. None of these studies establishes that altered completion of mitochondrial BER alone accounts for the observed metabolic or cellular phenotypes. Abbreviations: BER, base excision repair; HFD, high-fat diet; MPP^+^, 1-methyl-4-phenylpyridinium; mtDNA, mitochondrial DNA; NEIL1, Nei-like DNA glycosylase 1; NTHL1, NTH-like DNA glycosylase 1; OGG1, 8-oxoguanine DNA glycosylase 1.

**Table 3:** Noncancer disease models: observed outcomes and unresolved causal steps. Within each row, model systems and corresponding observations are presented in the order of the cited references.

Setting	Model and Intervention	Key Observation	Unresolved Causal Step	Ref.
Airway inflammation	TH5487 in mice; OGG1 S326C knock-in	Reduced induced inflammatory transcription; sustained promoter occupancy accompanied by increased NF-κB occupancy	Distinguish the contributions of DNA binding, ROS-dependent dimerization, and impaired catalysis	[1,2]
Antiviral interferon	NEIL2 at the RSV-responsive IFN-β promoter; OGG1 at RSV-responsive IFN-λ loci	NEIL2 limits early IFN-β transcription; OGG1 occupancy restricts IFN-λ expression	Define the locus-specific features that determine inflammatory versus antiviral transcriptional responses to the same enzyme	[21,45]
Telomeric senescence	Telomere-targeted 8-oxoG in human fibroblasts	Acute senescence requires OGG1- and MUTYH-dependent repair initiation; senescence occurs without substantial telomere shortening; sustained oxidation promotes telomere loss and crisis	Quantify repair completion during repeated oxidative stress and determine whether the same mechanism operates in nondividing cells	[4,49,50]
Lung and kidney fibrosis	TH5487 in lung injury; *Ogg1* interference in CKD; OGG1 inhibition in bleomycin lung	Reduced fibrogenic transcription; nuclear OGG1–pSmad3 association accompanied by attenuated renal fibrosis; altered macrophage polarization and mitophagy	Identify the responsible cell populations and determine whether a common molecular complex operates across organs	[24,25,44]
Atherosclerosis	Human aortic SMC; *Neil3* knockout in *Apoe*-deficient mice	Altered SMC phenotype; increased plaque burden accompanied by altered lipid handling	Identify the cell-autonomous substrate and establish the mechanistic relationship with Akt signaling	[30,31]
Diet-associated hepatic dysfunction	*Ogg1*-deficient mice with mitochondria-targeted hOGG1	Reduced mtDNA lesion burden and improved insulin sensitivity	Demonstrate causal mitochondrial repair flux and tissue-specific necessity	[3]
NEIL1-associated metabolism	Whole-body *Neil1* knockout; dietary challenge	Metabolic syndrome; diet- and genetic-background-dependent adiposity	Identify the initiating tissue and endogenous lesion	[27,28]
Aging brain	Mitochondria-targeted OGG1 in aging mouse and cell models	Reduced cytosolic mtDNA accompanied by lower STING-linked and hippocampal inflammatory readouts	Establish the mtDNA-sensing step and determine applicability to human aging	[48]
Neuronal vulnerability	MPTP-exposed *Neil1*-null mice; Pol β-reduced 3xTgAD mice; APE1-modulated rat hemorrhage model	Increased dopaminergic injury; worsened Alzheimer-like deficits; altered acute neuronal injury	Define the relevant lesion and extent of repair completion in each neural model	[56,61,67]
Neuroimmune and progenitor changes	MUTYH in Alzheimer-like mice; *Neil3* loss in Alzheimer-like and prion disease mice	Altered microglial activation, neurogenesis, and disease-stage behavioral phenotypes	Distinguish cell-autonomous effects and substrate identity from responses secondary to disease-associated stress	[60,62,65]

Notes: Entries summarize the outcomes directly measured in each model and should not be interpreted as establishing a complete disease mechanism. Species, cell type, and experimental system are specified because phenotypes observed in whole-body knockout models do not localize the relevant molecular reaction to a particular tissue or cell population. Abbreviations: 3xTgAD, triple-transgenic Alzheimer disease model; 8-oxoG, 8-oxo-7,8-dihydroguanine; APE1, apurinic/apyrimidinic endonuclease 1; CKD, chronic kidney disease; hOGG1, human OGG1; IFN, interferon; MPTP, 1-methyl-4-phenyl-1,2,3,6-tetrahydropyridine; mtDNA, mitochondrial DNA; MUTYH, mutY DNA glycosylase; NEIL1/2/3, Nei-like DNA glycosylases 1/2/3; NF-κB, nuclear factor κB; OGG1, 8-oxoguanine DNA glycosylase 1; pSmad3, phosphorylated Smad3; Pol β, DNA polymerase β; ROS, reactive oxygen species; RSV, respiratory syncytial virus; SMC, smooth muscle cell; STING, stimulator of interferon genes.

## Data Availability

No new data were created or analyzed in this study. Data sharing is not applicable to this article.

## Abbreviations

3xTgAD	Triple-transgenic Alzheimer disease
8-oxodG	8-oxo-7,8-dihydro-2′-deoxyguanosine
8-oxoG	8-oxo-7,8-dihydroguanine
AP	apurinic/apyrimidinic
APE1	apurinic/apyrimidinic endonuclease 1
BER	base excision repair
cGAS	cyclic GMP–AMP synthase
ChIP	chromatin immunoprecipitation
CKD	chronic kidney disease
cryo-EM	cryogenic electron microscopy
EXOG	exonuclease G
HFD	High-fat diet
IFN	interferon
IFN-β	interferon beta
IFN-λ	interferon lambda
IRF3	interferon regulatory factor 3
IRF7	interferon regulatory factor 7
MPP^+^	1-methyl-4-phenylpyridinium
MPTP	1-methyl-4-phenyl-1,2,3,6-tetrahydropyridine
mtDNA	mitochondrial DNA
MTH1	MutT homolog 1
MUTYH	mutY DNA glycosylase
NCP	nucleosome core particle
NEIL1	Nei-like DNA glycosylase 1
NEIL2	Nei-like DNA glycosylase 2
NEIL3	Nei-like DNA glycosylase 3
NF-κB	nuclear factor κB
NTH1	endonuclease III-like protein 1
NTHL1	NTH-like DNA glycosylase 1
OGG1	8-oxoguanine DNA glycosylase 1
PARP	poly(ADP-ribose) polymerase
PINK1	PTEN-induced kinase 1
Pol β	DNA polymerase β
PRC2	polycomb repressive complex 2
ROS	reactive oxygen species
RPA	replication protein A
RSV	respiratory syncytial virus
SARS-CoV-2	severe acute respiratory syndrome coronavirus 2
SMC	smooth muscle cell
SMUG1	single-strand-selective monofunctional uracil DNA glycosylase 1
STING	stimulator of interferon genes
UV-DDB	ultraviolet-damaged DNA-binding protein complex
XRCC1	X-ray repair cross-complementing protein 1
